# Anomalous Diffusion of Helium and Neon in Low-Density Silica Glass

**DOI:** 10.3390/membranes13090754

**Published:** 2023-08-24

**Authors:** Sergey V. Kukhtetskiy, Elena V. Fomenko, Alexander G. Anshits

**Affiliations:** 1 Federal Research Center “Krasnoyarsk Science Center SB RAS”, Institute of Chemistry and Chemical Technology SB RAS, Akademgorodok 50/24, Krasnoyarsk 660036, Russia; fom@icct.ru (E.V.F.); anshits@icct.ru (A.G.A.); 2 Department of Chemistry, Siberian Federal University, Svobodnyi pr. 79, Krasnoyarsk 660041, Russia

**Keywords:** low-density silica glass, expanded silica glass, helium, neon, permeability, selectivity, membrane separation of a helium–neon mixture

## Abstract

The diffusion properties of low-density non-porous silica glasses (expanded silica glasses) were researched with the aim of searching for the molecular structure of membrane materials intended for the effective separation of helium–neon gas mixtures. It has been shown on a large number (84) of computer models of such glasses that there are molecular structures of silica in which various helium and neon diffusion mechanisms are simultaneously implemented: superdiffusion for helium and subdiffusion for neon. This makes it possible to significantly (by 3–5 orders of magnitude) increase the helium permeability of such glasses at room temperature and maintain a high selectivity for the separation of helium and neon (at the level of $10^{4}$–$10^{5}$) at the same time.

## 1. Introduction

The task of efficient separation of a helium–neon mixture arises during the production of pure neon. Neon, a light inert gas, is widely used in modern technology and science. A well-known example is neon lamps. High-purity (99.999%) neon is the most important and practically irreplaceable component part of gas mixtures used in ultraviolet excimer lasers [1]. Lasers of this type have a lot of practical applications, such as photolithography within the nanometer range, laser weapons, thermonuclear fusion, submicron processing, and laser surgery [2]. Liquid neon is an important cryogenic coolant within the temperature range from 24 to 44 K [3,4]. This is due to the fact that it has a heat of vaporization 40 times greater than liquid helium and 3 times greater than liquid hydrogen, and it is safe, unlike the latter.

The Earth’s atmosphere is the only source of neon suitable for its industrial production. The volume content of this gas in the atmosphere is only 18 ppm. Therefore, the initial raw material for neon production is helium–neon concentrates obtained from non-condensable exhaust gases of large air separation plants of metallurgical industries [5,6,7]. The purified concentrate contains 75–77% neon and 25–23% helium. The content of impurities does not exceed 0.001%. Thus, the final stage of producing pure neon is limited to the task of separating the helium–neon mixture.

Since helium and neon are inert gases, such a separation may only be performed via physical methods. Methods such as distillation and sorption require low temperatures (30–78 K). This leads to significant operating costs [8], and as a consequence, the high price of pure neon. In this regard, non-cryogenic technologies for the separation of a helium–neon mixture represent an undoubted interest, in particular a membrane technology [9]. A comparative study of several helium–neon mixture separation technologies, including the membrane one, shows that the membrane technology has the minimum separation work, the maximum degree of thermodynamic perfection, and the maximum extraction factor [10]. Silica glass was considered as a membrane material—capillaries made of optical quartz produced by Gus-Khrustalny with an average density of 2.2 g/cm${}^{3}$.

Ordinary silica glass has high selectivity for the separation of a helium–neon mixture (5500 at 300 K), but helium permeability at room temperature is insufficient for industrial applications (only about $5\cdot 10^{-2}$ barrer) [11]. Increasing the process temperature makes it possible to increase the helium permeability, but leads to a sharp drop in selectivity.

To date, the structure of silica glass and the mechanisms of migration of light inert gases (He, Ne) in it have been fairly well studied [12]. Structural elements of silica glass are silicon–oxygen tetrahedra. At the center of the tetrahedron is a silicon ion, and at the vertices are oxygen ions. Glass itself under normal conditions is an random spatial network of silicon–oxygen tetrahedra connected by vertices. The silica glass network contains a large number of small voids where a dissolved helium atom (the number of such voids is about $2.3\cdot 10^{21}$ cm${}^{-3}$) or neon atom ($1.3\cdot 10^{21}$ cm${}^{-3}$) can be placed. These voids are called solubility sites. Adjacent solubility sites are separated from each other by walls consisting of siloxane rings. The siloxane rings contain a different number of SiO members with a distribution maximum near 6.

Migration of helium or neon in silica glass occurs as follows. Upon dissolution, an inert gas atom enters one of the solubility sites. An atom in the solubility site performs thermal vibrations as in a conventional potential well. With enough energy, this atom can overcome the potential barrier and jump to an adjacent solubility site through one of the siloxane rings. Then, the process is repeated. Due to the predominance of six-membered rings with a relatively high potential barrier in silica glass [13], the probabilities of helium and neon hopping differ markedly. It is this difference that ensures the high selectivity of the separation of the helium–neon mixture using silica glass.

Based on this mechanism of migration of atoms dissolved in silica glass, it can be assumed that an increase in the size (volume) of solubility sites, or equivalently, a decrease in glass density, will increase the glass permeability at low temperatures. Indeed, with an increase in the volumes of solubility sites, the solubility coefficient will increase. The length of jumps will also increase and the number of potential barriers per unit of the migration path of the dissolved atom will decrease. This will increase the diffusion coefficient. As a result, the permeability coefficient should also increase, equal to the product of the solubility coefficient and the diffusion coefficient.

On the whole, the problem of obtaining low-density silica glasses has long been solved both at the laboratory and at the industrial level. The density of such glasses is presented in a wide range—from a density close to the density of ordinary silica glass (dense microporous silica ceramics with a density of about 2.2 g/cm${}^{3}$) [14] to extremely expanded structures (aerogels with a density of several mg/cm${}^{3}$) [15]. Technologies for manufacturing membranes from such materials have also been developed. A brief review of the methods can be found in [16]. However, there are no experimental data on the separation of a helium–neon mixtures using membranes based on low-density silica in the open literature. There is also no information that would make it possible to theoretically evaluate the diffusion properties of existing low-density silica glasses for a helium–neon pair.

Thus, the question of the very existence of low-density silica structures, which would have a higher helium permeability compared to ordinary silica glass but at the same time without a significant loss in the selectivity of the separation of the helium–neon mixture, remains open. This theoretical study is devoted to clarifying this issue.

## 2. Materials and Methods

### 2.1. Generation of Computer Models of Expanded Silica Glass

The created set of computer models of low-density silica glass is designed to solve a very specific task—the search for molecular configurations of silica that are optimal from the standpoint of helium and neon mixture membrane separation. Therefore, many properties of the models being created, for example, thermodynamic or optical ones, may not be essential for this task. This gives sufficient freedom in choosing methods for creating such models and their molecular structure formation modes. It is sufficient that the following requirements be met:

1. The model should be an irregular spatial network of silicon–oxygen tetrahedra connected by vertices. The short-range order (i.e., the tetrahedra parameters) should correspond to the short-range order of a regular silica glass;
2. The model must be metastable. That is, the model atomic configuration must be locally stable and should be at a local energy minimum;
3. There should be no through porosity of the material. This requirement is necessary to implement the activation (jumping) nature of helium and neon diffusion in such a matrix and to ensure high selectivity in the separation of these gases.

Silica glass models that meet the above-mentioned requirements will be referred to as “expanded silica glass” below. It is possible to determine a parameter that characterizes the degree of glass expansion for each such model. This is the expansion coefficient $E=\left(\rho_{0}-\rho \right)/\rho_{0}$, where $\rho_{0}=2.2$ g/cm${}^{3}$ is the density of regular silica glass, ρ is the density of expanded silica glass. As for a fixed composition of the material, this parameter is equal to the relative variation in the porosity of silica during its expansion.

The recent studies of the silica glass compressibility in a helium atmosphere [17,18] have shown that at pressures of 1–10 GPa, the compressibility of samples in helium is significantly lower than when operating in other media. This goes to prove that helium effectively penetrates the interstitial space of silica. The estimates of the high-pressure helium solubility in silica glass made in [17] give values up to 1–2 mol He/mol SiO${}_{2}$. Such high solubility, when combined with high permeability, makes high-pressure helium an ideal agent (template) for modifying the structure of silica glass by increasing the volumes of solubility sites. These factors were taken as the basis for the methodology of obtaining models of expanded silica glass.

Computer models of expanded silica glass were obtained via molecular dynamics simulation of the process of silica melting and subsequent melt-quenching in a high-pressure helium atmosphere. The initial configuration of atoms was a cubic cristobalite containing 1000 silicon atoms and 2000 oxygen atoms. Helium (1000 atoms) was uniformly located in the cristobalite interstitial space. All atoms were located in a cubic computational cell with periodic boundary conditions. The initial pressure was 8 GPa, the temperature was 300 K. The dynamic simulation was fulfilled within the framework of the NPT ensemble. Therefore, the volume of the system, and consequently, the density of silica, were not fixed, but were obtained automatically during the simulation process.

**Figure 1:**
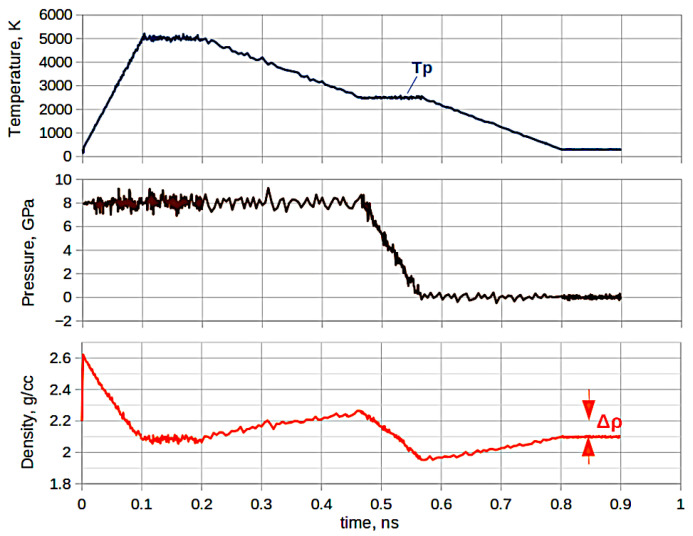
The chart of the process of generating expanded silica glass models via the method of molecular dynamics. The lower diagram shows the dynamics of the density of a pure silica matrix, excluding helium atoms.

A chart of a typical process for generating low-density silica glass models is shown in Figure 1. The two upper diagrams show the temperature and pressure in the system, and the lower diagram shows the density of silica (matrix) excluding helium atoms.

The system was heated to the homogenization temperature (5000 K) at a constant pressure during 0.1 ns at the first stage. The density of silica decreased due to heating and expansion of the system, as can be seen from the lower diagram. Then, the temperature and pressure were fixed during the next 0.1 ns. The silica melted with helium dissolved in it came to equilibrium. Then, the thermostat temperature was lowered to a certain temperature $T_{p}$ near the glass transition temperature $T_{g}$. This was the first stage of quenching.

The thermostat temperature was fixed after reaching $T_{p}$, and the barostat pressure was released to atmospheric pressure within 0.1 ns. When the pressure is released, the solubility of helium decreases, a supersaturation of the helium solution is created in silica, and a large number of nuclei of helium microbubbles appear, which subsequently form solubility sites of an increased volume compared to regular silica glass. Qualitatively, this stage of the process resembles the formation of pumice during volcanic eruptions.

The thermostat was further cooled to room temperature (the second stage of quenching) and the system was finally relaxed at atmospheric pressure and room temperature. In order for helium microbubbles in solubility sites to have a prominent influence on the structure of expanded silica glass, the quantity of dissolved helium atoms must be comparable with the quantity of glass structural elements. That is, the density of dissolved helium atoms should be of the order of the condensed phase (liquid helium) density. This explains the need to use high pressures in the process of generating models.

While varying the pressure release temperature $T_{p}$ with regard to the glass transition temperature $T_{g}$, one can control the final density of the glass, and accordingly, its expansion coefficient. In this study, the parameter $T_{p}/T_{g}$ varied within the range from 0.4 to 1.5, which ensured the obtaining of glass models with expansion coefficients from 0 to 25%. In total, 84 models of expanded silica glass were obtained in this way, which were used for calculating the permeability and selectivity. A more detailed description of the methodology set forth above and examples of the resulting glass structures can be found in the study [19].

Molecular dynamics simulation of the silica melting and hardening processes was fulfilled using the HOOMD-blue package [20] with a built-in pppm electrostatics module to speed up calculations on graphics processing units (GPUs) [21]. The methodology for modeling the silica melt, the formation of silica glass, and the potentials used for silicon and oxygen ions are described in the study [22]. The effective potentials of the interaction of helium atoms with silicon–oxygen tetrahedra were taken from [23].

### 2.2. Calculation of Solubility, Diffusion, and Permeability Coefficients

The calculation of the diffusion characteristics of each model began with the construction of a discrete three-dimensional potential landscape for each probe particle (helium or neon atom). The model space was divided into a set of equal cells (voxels). At the center of each cell, the potential energy of the probe particle was calculated. The effective interaction potentials of probe particles (helium and neon) with silicon–oxygen tetrahedra of matrix were taken from [23]. The resulting three-dimensional array (cuboid) of voxels with calculated potential energy values is a discrete potential landscape of the model for a given probe particle. In this work, cuboids of 256 × 256 × 256 voxels were used. Voxel sizes are 0.14–0.15 Å. Such a spatial resolution is quite sufficient for analyzing the topology of free space and calculating the diffusion characteristics of models. A more detailed description of the procedure for constructing such potential landscapes can be found in [24].

After constructing potential landscapes according to the technique described in [25], the solubility coefficients of helium and neon were calculated for each model of expanded silica glass at a temperature of 300 K.

To estimate the diffusion coefficients *D* of dissolved gases, the dependence of the mean squared displacement of a probe particle $\langle \Delta r^{2}\rangle =6\cdot D\cdot t$ on time *t* (Einstein’s formula) is usually used. But two features arise when modeling the low-temperature migration of probe particles in sufficiently rigid matrices of expanded silica glass.

First, the probabilities of probe particles jumping between adjacent solubility sites rapidly decrease with decreasing temperature. That is, jumps become “rare events”. Therefore, the vast majority of calculations of the dynamics of a probe particle will fall on the simulation of the less informative motion of a particle inside the solubility sites. As a result, collecting jump statistics for a correct estimate of the diffusion coefficients becomes problematic. The method based on the transition state theory [26,27] is one of the effective ways to model such rare events. The methodology described in [28] was used to estimate the diffusion coefficients. Its essence lies in the transition from a detailed simulation of probe particle dynamics in real space to a statistical description of jumps between adjacent sites of probe particle solubility in a matrix. Solubility sites are local minima of the potential landscape of a probe particle. The attraction domains of all local minima of the potential landscape unambiguously divide the space into separate cells divided by surfaces containing saddle points of adjacent local minima. Further, the transition state theory methods are used to calculate the average lifetimes in cells and the probabilities of jumps between adjacent cells. Ultimately, instead of a detailed calculation of the real dynamics of a probe particle in cells, one can proceed to model random walks of a probe particle over a network of solubility sites with given average lifetimes of a particle at nodes and probabilities of jumps to adjacent nodes. Simulation of random walks was fulfilled via Monte Carlo methods. Such a rough, “coarse-grained” approach makes it possible to effectively calculate the time dependence of the mean-squared displacement of a probe particle, and thus estimate the diffusion coefficients at low temperatures.

The second feature of the migration of test particles in dense and rigid matrices is that the dependence of the mean-squared displacement on time may deviate from a linear one. That is, the so-called ‘anomalous diffusion’ can be realized [29,30]. Usually, such diffusion modes are described by the relation

(1) $$\langle \Delta r^{2}\rangle =K\cdot t^{\alpha },$$

where the α determines the nature of diffusion. For α<1, subdiffusion takes place; for α>1, superdiffusion takes place. In this study, the indicator α was calculated by power–law regression of the initial sections of the $\langle \Delta r^{2}\rangle$ dependence on *t*.

In the case of long random walk times, with due regard for periodic boundary conditions, the dependence of $\langle \Delta r^{2}\rangle$ on *t* straightens out, the nature of diffusion approached normal, and the diffusion coefficient was determined from the slope of the dependence of $\langle \Delta r^{2}\rangle$ on *t* in these time periods.

The permeability coefficient was calculated by multiplying the solubility coefficient by the diffusion coefficient.

### 2.3. Calculation of the Percolation Energy of Helium and Neon Atoms

In this study, this parameter was used to control the presence of through porosity in the sample. The calculations were made as follows. First, the sample was centered on the cavity with the maximum volume, and a discrete potential landscape of the probe particle (helium or neon atom) was created as described in Section 2.2.

Then, a connected set of voxels (cluster) was determined that could be reached by a probe particle moving from the center with specified energy. To build such a cluster, the “wave” method [31] was used, which, in fact, implements the Huygens principle in the discrete voxel space of a potential landscape. If the probe particle did not reach the boundaries of the computational cell, then its energy increased and the cluster building procedure was repeated.

The cluster reached one or another boundary of the computational cell at a certain energy of the probe particle, that is, it became a percolation one. The minimum energies of formation of percolation clusters averaged over the entire boundaries were used as an estimate of the percolation energy.

## 3. Results

The average coordination numbers of silicon ions for all 84 models are close to 4 and are 3.993 ± 0.004 for all models. Thus, the majority of silicon ions are located at the centers of silicon–oxygen tetrahedra. The average coordination number of oxygen ions is 1.997 ± 0.002. This suggests that most of the oxygen ions are bridged ones. The average Si-O bond length is 1.624 ± 0.001 Å. Thus, the structural elements of the expanded silica glass models are close to the structural elements of regular silica glass. That is, the first requirement for the parameters of structural elements of expanded silica glasses (see Section 2.1) is met.

**Figure 2:**
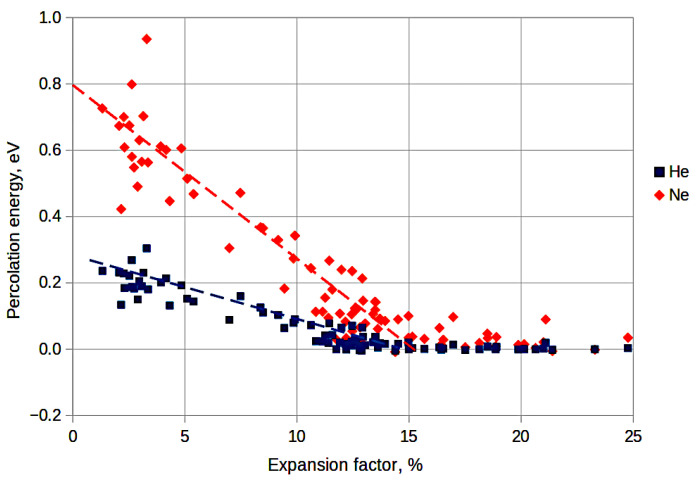
Dependence of the percolation energy of helium and neon atoms on the glass expansion coefficient.

The second requirement of Section 2.1 lies in the metastability of the model molecular structures. It is performed automatically due to the fact that the model structure formation process and the structure final relaxation were fulfilled via the molecular dynamics method within the framework of the NPT ensemble. To fulfill the third condition from Section 2.1 (absence of through porosity), let us consider the percolation energies of the models for helium and neon. The dependencies of this energy on the expansion coefficient of silica glass are shown in Figure 2.

It can be seen from the figure that the percolation threshold decreases linearly up to an expansion coefficient of 14–15%, and then it remains close to zero. That is, helium and neon atoms do not have to overcome any substantial potential barriers when moving in the samples with expansion coefficients of more than 15%. This may indicate the possible presence of through pores in such samples. Although, there is definitely no through porosity in the models with expansion coefficients less than 15%. That is, all three conditions listed in Section 2.1 are met for these models.

**Figure 3:**
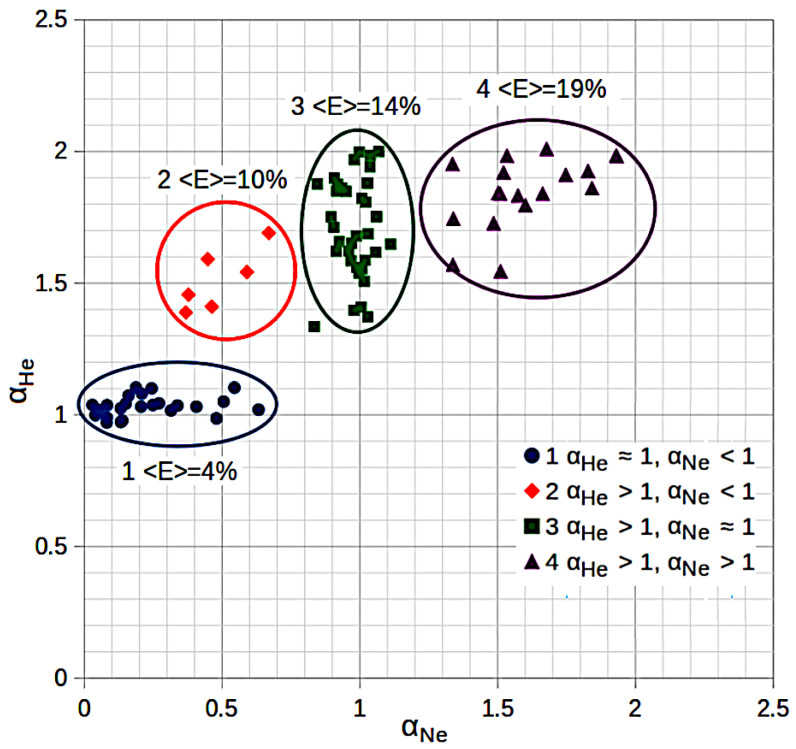
Cluster analysis of models based on $\alpha_{He}$ and $\alpha_{Ne}$ exponents. 〈E〉 means the group-average glass expansion coefficients.

A more detailed screening of expanded silica models in their diffusion properties may be seen in Figure 3. In this diagram, the samples are located in the space $\alpha_{He}-\alpha_{Ne}$, where $\alpha_{He}$ and $\alpha_{Ne}$ are the growth exponents of the mean squared displacement with time for helium and neon, respectively (see Equation (1)).

According to the results of the cluster analysis (Figure 3), it can be seen that the models are clearly divided into four groups by the nature of helium and neon diffusion.

Group 1—normal diffusion of helium ($\alpha_{He}\approx 1$) and anomalous diffusion of neon by subdiffusion type ($\alpha_{Ne}<1$). The group-average expansion coefficient is 4%, the percolation energy of helium is 0.187 eV, and that of neon is 0.58 eV.

Group 2—helium demonstrates an anomalous diffusion mode of the superdiffusion type ($\alpha_{He}>1$), while neon continues to migrate in the subdiffusion mode ($\alpha_{Ne}<1$). The group-average expansion coefficient is 10%, the percolation energy of helium is 0.086 eV, and that of neon is 0.297 eV.

Group 3—helium continues to migrate in the superdiffusion regime ($\alpha_{He}>1$), while neon passes from subdiffusion to the normal diffusion mode ($\alpha_{Ne}\approx 1$). The group-average expansion coefficient is 14%, the percolation energy of helium is 0.023 eV, and that of neon is 0.102 eV.

Group 4—both gases migrate in the superdiffusion mode ($\alpha_{He}>1,\alpha_{Ne}>1$). The group-average expansion coefficient is 19%, the percolation energy of helium is 0.002 eV, and that of neon is 0.029 eV.

**Figure 4:**
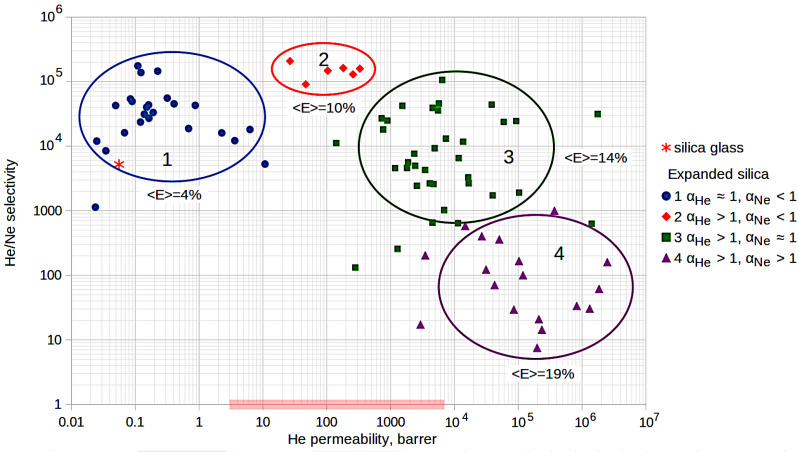
Dependence of He/Ne selectivity on helium permeability for 84 models of low-density silica glass.

The influence of the silica glass expansion coefficients and different diffusion modes of helium and neon on the selectivity of separation of these gases and permeability can be conveniently represented in the form of a Robeson diagram [32], which was plotted for all 84 models of expanded silica glass used in this study and is shown in Figure 4. The division into groups corresponds to the previous diagram (Figure 3). To compare the helium permeability of expanded silica glasses with the helium permeability of typical polymer membranes, the red rectangle on the horizontal axis shows the range of helium permeability of polymer membranes. The bounds of the rectangle were calculated based on the data taken from [32]. The values 〈E〉 near the groups of samples mean the group-average coefficients of the glass expansion. The red asterisk on the left side of the diagram corresponds to regular silica glass (calculated from [11]).

It can be seen from the diagram in Figure 4 that the samples of group 1 (slightly expanded glasses <E> = 4%) have comparable (and somewhat higher) values of selectivity and permeability compared to conventional silica glass (red asterisk).

Group 2 samples (<E> = 10%) demonstrate high selectivity of helium and neon separation (at the level of $10^{5}$), and at the same time, good helium permeability at room temperatures—at the level of typical polymer membranes. Such glasses are still poorly permeable to neon (subdiffusion), but are already well permeable to helium (superdiffusion).

The helium permeability of group 3 samples (<E> = 14%) is higher than the permeability of polymer membranes on average, but at the same time, the selectivity of He/Ne remains at a sufficiently high level (about $10^{4}$ on average). This is due to the fact that the superdiffusion mode is implemented for helium, and the normal diffusion mode is implemented for neon in these samples.

Glasses of the 4th group (<E> = 19%) are well permeable to both helium and neon. Both gases demonstrate superdiffusion. Their helium permeability is much higher than the permeability of polymer membranes, but the selectivity is already significantly lower (at the level of 10–100 on average) compared to samples of other groups.

## 4. Discussion

Tetrahedral structural elements are capable of forming a wide variety of metastable structures that differ in the packing geometry of structural elements. These structures determine the free space topology of the resulting materials, and consequently, their diffusion properties with regard to the gases dissolved in them. That is why the choice of silica as the base material is a good choice in terms of generating as much variety of molecular structures as possible in the resulting models. The use of molecular dynamics automatically rejects unstable tetrahedral structures when generating models within NPT ensembles. The presence of a large number of dissolved helium atoms makes it possible to shift the formation of target structures in a desired direction, i.e., to increase the volume of cavities (solubility sites) in the models.

A large number (84) of the generated models with a variety of free space geometry made it possible to fulfill a quite effective screening of models in terms of their diffusion properties with regard to helium and neon. As a result of screening, two groups of models (groups 2 and 3) were identified, which can make it possible to formulate structural requirements for membrane materials in order to efficiently separate helium–neon mixtures.

It has to be noted that the production of such materials in practice should not necessarily be associated with such hard synthesis conditions that were used during the generation of computer models. These conditions are stipulated only by the desired depth of silica glass modification in order to obtain the maximum variety of molecular structures used in screening for diffusion properties. Moreover, these materials do not have to be silica-based as long as they are designed to separate pure inert gases at room or low temperatures. That is, such a high chemical and thermal stability of the membrane material as silica is not mandatory. The necessary condition is to implement such a molecular structure of the material, wherein various mechanisms of diffusion of the separated gases are simultaneously implemented, specifically superdiffusion for the light component (helium in this case) and subdiffusion for the heavy component (neon).

## 5. Conclusions

1. Eighty-four computer models of expanded silica glass were generated with an expansion coefficient ranging from 0 to 25% via molecular dynamics simulation of the process of quenching a silica melt in a high-pressure helium atmosphere;
2. Screening of these models based on their diffusion properties with regard to helium and neon makes it possible to distinguish four groups, two of which demonstrate high helium permeability (from 10 to $10^{5}$ barrer) with high selectivity of helium and neon separation ($10^{4}$–$10^{5}$);
3. Such high parameters are due to the fact that various mechanisms of diffusion of the gases being separated are implemented simultaneously in these models: superdiffusion for helium and sub- or normal diffusion for neon. The structural features of such models may be useful while designing real membranes for the efficient separation of helium and neon.

## Data Availability

The data are available from the corresponding author upon request.
